# The Development of Toad Toxins as Potential Therapeutic Agents

**DOI:** 10.3390/toxins10080336

**Published:** 2018-08-20

**Authors:** Ji Qi, Abu Hasanat Md Zulfiker, Chun Li, David Good, Ming Q. Wei

**Affiliations:** 1 Menzies Health Institute Queensland and School of Medical Science, Griffith University, Gold Coast, QLD 4222, Australia; ji.qi2@griffithuni.edu.au (J.Q.); ivy_sampras@hotmail.com (C.L.); david.good@acu.edu.au (D.G.); 2 Department of Biomedical Sciences, Marshall University, Huntington, WV 25701, USA; zulfikermsgu@outlook.com; 3 School of Physiotherapy, Australian Catholic University, Banyo, QLD 4014, Australia

**Keywords:** toad toxins, Chansu, Huachansu, cane toad, bufadienolides, indolealkylamines, inflammation, cancer, obsessive–compulsive disorder (OCD)

## Abstract

Toxins from toads have long been known to contain rich chemicals with great pharmaceutical potential. Recent studies have shown more than 100 such chemical components, including peptides, steroids, indole alkaloids, bufogargarizanines, organic acids, and others, in the parotoid and skins gland secretions from different species of toads. In traditional Chinese medicine (TCM), processed toad toxins have been used for treating various diseases for hundreds of years. Modern studies, including both experimental and clinical trials, have also revealed the molecular mechanisms that support the development of these components into medicines for the treatment of inflammatory diseases and cancers. More recently, there have been studies that demonstrated the therapeutic potential of toxins from other species of toads, such as Australian cane toads. Previous reviews mostly focused on the pharmaceutical effects of the whole extracts from parotoid glands or skins of toads. However, to fully understand the molecular basis of toad toxins in their use for therapy, a comprehensive understanding of the individual compound contained in toad toxins is necessary; thus, this paper seeks to review the recent studies of some typical compounds frequently identified in toad secretions.

## 1. Introduction

Toad toxins from parotoid or skin glands have significant therapeutic value for a plethora of diseases [1]. In China and other East and Southeast Asian countries, toad toxins traditionally refer to the processed and dried venom from parotoid glands of the toad *Bufo bufo gargarizans* [2]. In traditional Chinese medicine (TCM) it is known as Chansu, while in Japan it is known as Senso, which has been recorded since the Tang Dynasty (618–907 B.C.) [3]. These products have been used for treating pain and inflammatory diseases with more than a dozen remedies on the market [4]. Similarly, the water extracts from the skins of *B. b. gargarizans* is known as Huachansu (Cinobufacini), which was developed in China about 20 years ago, and had been successfully used to treat various types of cancers with low toxicity and few side effects [5,6]. Both molecular and clinical data have revealed the chemical constituents, as well as the mechanisms of action from their use [7,8]. Although different groups of constituents may have diverse functions, it is well known now that bufadienolides, such as bufalin and cinobufagin, are considered as the main bioactive compounds in toad toxins. These groups of compounds are C-24 steroids with similar properties as cardiac glycosides medications such as digoxin. The pharmaceutical use of bufadienolide is primarily considered as a Na^+^/K^+^-ATPase inhibitor for treating congestive heart failure and arterial hypertension, due to its property of high binding affinity to phosphoenzyme [9,10]. However, there have been reports indicating that an overdose of cardiac glycosides may cause prolonged blockage of Na^+^/K^+^-ATPase in these cells, resulting in cardiac arrest [11]. Recent studies have also revealed the therapeutic potential of bufadienolides in immunomodulation, anti-inflammation, and anti-neoplastic activity [12,13]

It has also been found that ancient people of Mesoamerica had used toads, *B. marinus* or *B. alvarius*, as a hallucinogen via licking toad skins directly, or smoking the prepared powder [14]. Studies have shown that indolealkylamines (IAAs) in toad skin, primarily bufotenine, are responsible for these hallucinogenic effects [15]. IAAs are biogenic amines and derivatives of 5-hydroxytryptamine, producing their effects through binding of serotonin receptors [3]. Due to the hallucinogenic effect, the use of bufotenine has increased in New York, USA in last century, and has drawn the attention of scientists to study the potential of bufotenine for the treatment of neuropsychiatric disorders [16].

Past significant studies have primarily focused on Chansu and Huachansu, due to their likely effect on cancer treatment. Recent studies have increasingly examined the therapeutic potential of other species of toads. An example of this is the studies of Australian cane toads (*B. marinus*), which originated from North American, but were introduced into Australia in 1935 to control cane beetles. The cane toads have become a biological and environmental disaster in northern Australia, due to their fast reproduction speed and lack of natural predators [17]. There are numerous scientists who have now started to consider the pharmaceutical potential of these cane toads [18,19,20]. In a recent study, the umbilical arteries isolated from human fetal placentas have been used as a model in studies comparing the cardiac glycoside-like activity of cane toad skin extracts prepared in different extraction procedures [18]. The inhibitory effect of cane toad skin aqueous extracts (CTSAE) on Na^+^/K^+^-ATPase was also demonstrated in other experimental models [18]. In our laboratory, we have recently shown the anti-inflammatory effect of CTSAE via inhibiting the release and expression of TNF-α and IL-6, and the suppression of nuclear factor (NF)-kappa (κ)B in vitro [19]. A further study from us has also indicated that CTSAE enhanced the expression of 5-HT2AR and D2R, with the modulation of Gq/11-PLCβ signaling pathway and c-FOS transcription factor, which may improve the therapeutic effect on certain diseases, such as obsessive–compulsive disorder (OCD) [20].

Previous reviews are mostly focused on the therapeutic effects of whole extracts, such as Chansu and Huachansu. Thus, we believe that it is important to understand the effect of individual compounds, which would enable us to explore the development of toad toxins as medicines.

## 2. Chemicals Components in Different Species of Toads

Several classes of compounds have been identified from the parotoid or skins glands of toads, including peptides, steroids, indole alkaloids, bufogargarizanines, organic acid, and others [2,21,22,23,24,25]. Bufadienolides and indolealkylamines are considered as the two main groups of compounds with therapeutic potential (Table 1) [26,27].

A previous study has investigated the toad venoms from different *Bufo* species, in which 43 compounds were identified in the methanolic extracts of the different samples. Gamabufotalin, arenobufagin, telocinobufagin, bufotalin, cinobufotalin, bufalin, cinobufagin, and resibufogenin, were identified as major constituents of Chansu. Low levels of resibufogenin, but no cinobufagin was observed in the samples from *B. melanosticus*, *B. marinus*, and *B. viridis*. Three compounds, telocinobufagin, marinobufagin, and bufalin, were found in all samples [2]. These results have been confirmed by other studies using different analytical methods [28,29]. The indolealkylamines in Chansu have been analyzed in another study, including bufotenine, bufotenidine, bufobutanoic acid, serotonin, bufotenine *N*-oxide and *N*-methyl serotonin were also identified [30].

The chemical constituents of bufadienolides and indolealkylamines have also been identified in Huachansu. There were eight bufadienolide compounds, including bufalin, cinobufagin, recinobufagin, cinobufotalin, telocinobufagin, gamabufotalin, arenobufagin, and bufotalin, which were detected in an injected preparation of Huachansu from a previous study [31]. Additionally, the indolylalkylamines—including bufotenine, bufotenidine, cinobufotenine, and serotonin—were found in Huachansu, as mentioned in previous literature [32,33].

Several studies have evaluated the chemical compounds in other species of toads, such as cane toads collected from sites in Australia. They found that cane toad parotoid gland secretion contains bufadienolides, including high levels of marinobufagin; medium levels of bufalin, telocinobufagin, arenobufagin, and marinobufotoxin; low levels of resibufogenin, hellebrigenin, marinobufagin-3-pimeloyl-l-arginine ester, bufalin-3-pimeloyl-l-arginine ester, and bufalitoxin; and detectable levels of many other biotransformed bufadienolides [34]. A recent study performed by us, using high-performance liquid chromatography coupled with a hybrid quadrupole-time of flight mass spectrometer (HPLC/MS-Q-TOF), examined the chemicals in secretions of the cane toad parotoid glands. We found the presence of twelve key chemicals in the secretion, including several major bufadienolides, which was further confirmed by calculating the exact differences between the theoretical and measured mass of each assumed compound [35]. Following this study, a similar analytical method was used in our laboratory to assess the chemicals in extracts from cane toad skin, and up to 42 constituents, including both bufadienolides and indolylalkylamines, were identified [22].

Other numerous studies have been carried out in different species of toads, such as *B. melanosticus* from different regions [24,25,36,37]. Taken together, these data have provided us with the chemical profiles of toad toxins, which are essential for the study of their pharmaceutical effects.

## 3. The Bioactivity Studies of Bufadienolides

**Figure 1:**
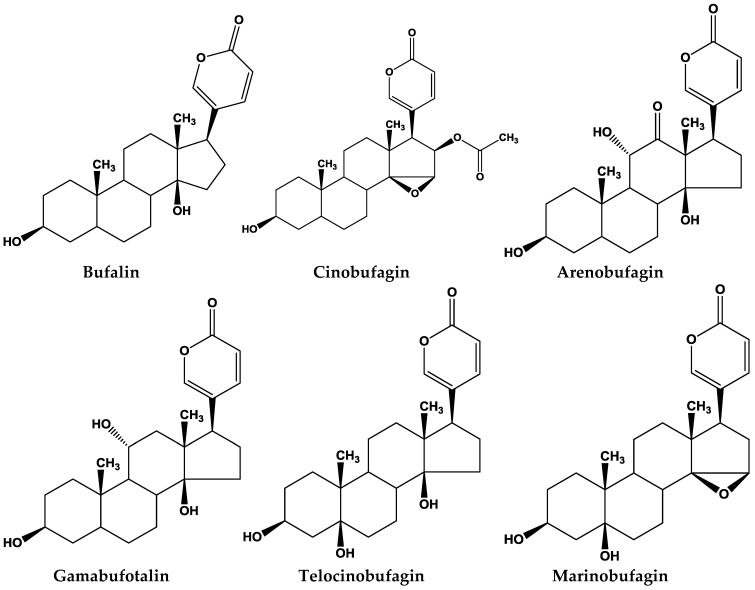
Major bufadienolides found in toad species.

The potential pharmaceutical effects of bufadienolides contained in toad toxins have been studied in recent years. Several in vitro studies have demonstrated that they have predominant effects on the inhibition of different tumor cell growth, inducing cell cycle arrest, apoptosis, and in regulating the expression of malignant related genes/proteins in human cancer cells [38,39,40,41] (Table 2). Here, we reviewed and listed the major compounds from some of the major studies (Figure 1).

### 3.1. Bufalin

Bufalin is a major compound in Chansu, Huachansu, as well as the toxins of other toad species, such as *B. marinus*. Several studies have demonstrated its anti-inflammatory and anticancer effects through inhibiting NF-κB pathway, which is a crucial pathway in both anti-inflammation and cancer [42,43]. The effect of bufalin on the treatment of the asthmatic response has been studied in a murine model. The mouse asthma model was developed by ovalbumin (OVA)-induced BALB/c mice. The results demonstrated that bufalin reduces hyperresponsiveness, and inhibits the OVA-induced activation of inflammatory cells, including macrophages, eosinophils, lymphocytes, and neutrophils and cytokines, including IL-4, IL-5, and IL-13. Histological staining examined the reduction of inflammatory cell infiltration and goblet cell hyperplasia, while the blockage of NF-κB was evaluated by Western blot [44]. The anti-inflammatory and analgesic effects of bufalin have also been studied in a carrageenan-induced paw oedema model. Bufalin downregulated the expression of nitric oxide synthase (iNOS), cyclooxygenase-2 (COX-2), interleukin-1β (IL-1β), interleukin-6 (IL-6), and tumor necrosis factor-α (TNF-α), to which the inhibitory effect on the master switch of NF-κB signaling is attributed [45].

The antimetastatic effect of bufalin was studied in human hepatocellular carcinoma SK-Hep1 cells to determine if bufalin plays an important role in mortality of cancer patients, in which the expression of matrix metalloproteinases (MMPs), such as MMP-2 and -9 are inhibited, while phosphoinisitide-3-kinase (PI3K) and phosphorylation of AKT are reduced with the suppression of NF-κB [46]. Another study has shown the antimetastasis effects of bufalin on NCI-H460 lung cancer cells, with similar mechanisms [47].

The anticancer property of bufalin has been validated in a wide range of cancer cells, including leukemia, prostate, gastric, liver, and breast. Studies have indicated that bufalin inhibits tumor growth through the induction of programed cell death via multiple pathways [48].

In a study using an animal model, bufalin has been shown to suppress the growth of BEL-7402 cells, human hepatocellular carcinoma (HHC) cells, in an orthotopic transplantation tumor model in nude mice [49]. This study has also shown bufalin-induced apoptosis in a tumor model by activating Bax without causing apparent toxicity [49]. In another study, nude mice injected with HCCLM3-R cells were studied after treatment with bufalin. Significant antitumor activities, and the reduction of the metastatic growth with the inhibition of AKT/GSK3β/β-catenin/E-cadherin signaling pathways, were found [50]. A study has also investigated anticolorectal cancer (CRC) effects of bufalin in HCT116 orthotopic xenograft model in mice. The results have indicated that bufalin inhibits tumor growth by inducing cell apoptosis through the intrinsic apoptotic pathway [51]. A human lung cancer cell line, NCI-H460, injected into a BALB/C nu/nu mouse model, was also studied after bufalin treatment, confirming a reduction in tumor size without significant drug-related toxicity [52].

### 3.2. Cinobufagin

Cinobufagin from toad *B. b. gargarizans* is known as the second major compound in Chansu and Huachansu; however, it is not detected in some other species of toads, such as Australian cane toad.

In a previous study, we have demonstrated that cinobufagin inhibited the growth of colon, prostate, skin, and lung cancers, in vitro. Specifically, cinobufagin induced apoptosis of HCT116 and HT29 via the caspase-3-dependent and -independent pathway, respectively. The inhibition of hypoxia-inducing factor-1 alpha subunit 75 has been demonstrated both in vitro and in vivo [53]. Further study has shown that cinobufagin inhibited the expression of cortactin in HCT116 cells, and HCT116 xenograft tumors in nude mice in vivo [54].

A study has also investigated the potential anti-osteosarcoma (OS) effect and the mechanisms of action of cinobufagin. The in vitro studies have indicated that cinobufagin induced the cell cycle arrest and apoptosis in OS cells with the involvement of Notch pathway suppression. Moreover, in the in vivo xenograft OS mouse model, cinobufagin inhibited OS cell growth with a suitable drug tolerance [55].

### 3.3. Arenobufagin

Arenobufagin has been shown to act against the growth of esophageal squamous cell carcinoma (ESCC) by triggering the activation of p53 through its phosphorylation, and caspase through intrinsic and extrinsic pathways both in vitro and in vivo. This study has also shown the selective effect in killing tumor cells and low toxicity toward Het-1A human normal esophageal squamous cells. Transfection of cells with p53 small interfering RNA can reverse this effect. Moreover, in vivo studies have confirmed the anticancer effect of arenobufagin by inhibiting the tumor growth through activation of the p53 pathway [56].

Arenobufagin has also shown anti-neoplastic activity against HCC HepG2 cells, as well as the corresponding multidrug-resistant HepG2/ADM cells, increasing Bax/Bcl-2 expression ratio, and inhibiting the phosphatidylinositol 3-kinase (PI3K)/Akt/mammalian target of rapamycin (mTOR) pathway. Arenobufagin inhibited the growth of HepG2/ADM xenograft tumors, which were associated with poly (ADP-ribose) polymerase cleavage, light chain 3-II activation, and mTOR inhibition [57].

Another study has demonstrated an antimetastasis and epithelial–mesenchymal transition (EMT) inhibitory effect of arenobufagin in PC3 cells by suppressing β-catenin. These results are also verified in a xenograft tumor mouse model [58].

Arenobufagin has also been shown anti-angiogenic activity through inhibiting vascular endothelial growth factor (VEGF)-induced viability, migration, invasion, and tube formation in human umbilical vein endothelial cells (HUVECs). Additionally, this effect has been confirmed via an in vivo model. Computer simulations suggested that arenobufagin interacted with the ATP-binding sites of VEGFR-2 by docking. Furthermore, arenobufagin inhibited VEGF-induced VEGFR-2 autophosphorylation, and suppressed the activity of VEGFR-2-mediated signaling cascades [59].

### 3.4. Gamabufotalin

There has been a study showing that gamabufotalin plays a role in angiogenesis inhibition through the blockage of VEGF-induced HUVEC proliferation, migration, invasion, and tubulogenesis. This study also demonstrated the effect of gamabufotalin in decreasing vessel density in human lung tumor xenograft implanted in nude mice, while inhibiting vascularization in matrigel plugs impregnated in C57/BL6 mice. Further studies, including computer simulations and Western blot analysis, have revealed that gamabufotalin interacted with the ATP-binding sites of VEGFR-2 using molecular docking. Furthermore, Western blot analysis indicated that the inhibitory effect of gamabufotalin for angiogenesis was due to the suppression of the VEGFR-2 signaling pathway [60].

The therapeutic potential of gamabufotalin in human multiple myeloma (MM) cells has also been studied. Results have shown that gamabufotalin inhibited cell growth and induced apoptosis via the activation of the ubiquitination process of c-Myc. The anticancer effect and inhibition of MM-induced osteolysis of gamabufotalin were further validated in a xenograft mouse model and SCID-hu model, separately [61].

Gamabufotalin has also shown effect in blocking the NF-kB pathway. A study has shown gamabufotalin strongly suppressed COX-2 expression by inhibiting the phosphorylation of IKKβ via targeting the ATP-binding site, which in turn, prevents NF-κB binding and p300 recruitment to COX-2 promoter in a range of human NSCLC, H1299, A549, H322, and H460 cell lines. In in vivo studies, gamabufotalin suppressed the tumor weight and size with the decreasing protein levels of COX-2 and phosphorylated p65 NF-κB in the tumor tissues of xenograft mice [62].

### 3.5. Other Key Bufadienolides

The immunoregulatory effect of telocinobufagin, another major compound in Chansu, was studied in vitro. The activation of several cytokines and immunocytes was observed [63]. Telocinobufagin and marinobufagin isolated from skin secretions of the Brazilian toad *B. rubescens* have been shown to exhibit antimicrobial activity inhibitory action over *Staphylococcus aureus* and *Escherichia coli* [23]. Differently from telocinobufagin, marinobufagin is a minor constituent in Chansu and Huachansu. However, it has been identified as the main component in the toxins of cane toads. Currently, there are still very few functional studies of marinobufagin.

## 4. Indolealkylamines

Indolealkylamines (IAAs) are known as derivatives of 5-hydroxytryptamine (5-HT), which primarily affect the central nervous system (CNS). To date, at least fourteen IAAs, including 5-methoxy-*N*,*N*-dimethyltryptamine (5-MeO-DMT) have been characterized; among these, bufotenine, bufotenidine, and cinobufotenine have been identified in the skins of toad species. Some IAAs are clinically used as antimigraine therapies, whereas the misuse of these chemicals may cause drug abuse. Recently, IAAs in toad toxins are considered as potential therapeutic compounds in developing new agents for treating several neurologic disorders, such as schizophrenia, depression, anxiety, obsessive–compulsive disorders, and chronic pain conditions, due to their potential 5-HT2A receptor selectivity in the CNS [49].

**Figure 2:**
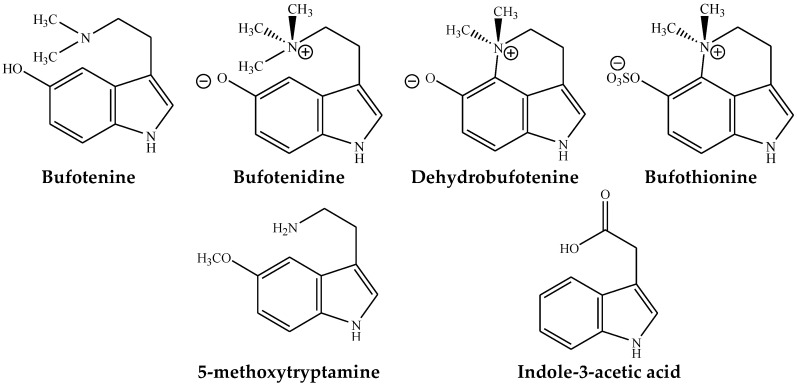
Major indolealkylamines found in toad species.

Some of the major IAAs found in toad toxins having pharmaceutical values, which are summarized below (Figure 2).

### 4.1. Bufotenine

Bufotenine was first identified from Senso in Japan, and Chansu in China [64]. Bufotenine binds to the 5-HT2A receptor in vitro, with a similar affinity to that of 4-bromo-2,5-dimethoxy-phenylisopropylamine (DOB) [65,66]. For many years, the activity of bufotenine remained a controversy, as to whether it was a hallucinogen or psychotomimetic. Though there are few reports about the significant pharmaceutical value of bufotenine, it was found to have potent psychotropic properties, and other psychotic symptoms, due to the similar physiological and structural features to lysergic acid diethylamide (LSD) in the 5HT2 receptor [67,68]. Bufotenine was also reported to be used as a biomarker in the diagnosis of various psychiatric disorders, such as schizophrenia and autism [69]. Recently, bufotenine isolated from the parotoid gland secretions of *Bufo bufo* was also reported to have cholinergic properties in α7 nicotinic acetylcholine receptors [70].

### 4.2. Bufotenidine

Like bufotenine, bufotenidine was also isolated from Senso in Japan and Chansu in China [65]. Bufotenidine was obtained from the skin of *Leptodactylus vilarsi melin*, which was found to have a hypertensive effect [71]. Bufotenidine showed marked neuromuscular blocking activity by producing the characteristic head drop in rabbits in doses of 5.2 ± 0.9 mg/kg iv. It also showed potent ganglionic stimulation and significant cholinergic-like action [72]. Recently, bufotenidine was isolated from the parotid gland secretions of *Bufo bufo* and reported to have cholinergic properties in α7 nicotinic acetylcholine receptors [71].

### 4.3. Dehydrobufotenine

Dehydrobufotenine was isolated from parotoid glands and skins of many species of toads, such as *B. marinus*, *B. arenarum*, and *B. b. gargarizans*, as the principal indolealkylamine [25,73]. Dehydrobufotenine was reported to show potent in vitro cytotoxicity against human tumor cell lines that were thought to act as DNA topoisomerase II inhibitors [74,75]. Additionally, dehydrobufotenine was used as a dry powder inhaler (DPI) in preparation of an antitumor drug for treating lung tumor [76].

#### 4.3.1. Bufothionine

Bufothionine was found in the skin of various toad species [25]. Recently, it was identified in Cinobufacini injection [77] and the skin of *B. b. gargarizans* [73,78]. Bufothionine was reported to inhibit the proliferation of human hepatocellular carcinoma cell lines [77]. Bufothionine was also reported to have cytotoxic activity against the murine leukemia cell line P388, and human hepatocellular carcinoma cell lines SMMC-7721 and BEL-7402 [79]. A new formulation of this alkaloid after isolation from toad skin has already been used for cancer therapy [80]. Bufothionine powder for inhalation was found in medicine for the treatment of pulmonary neoplasm [81].

#### 4.3.2. Other Indolealkylamines

Though there are at least 14 IAA identified in various toad species, very few of them were found to have significant pharmaceutical value, except the above four described here. Among others, 5-methoxytryptamine was reported to have antioxidant and radioprotective effects in various biological systems [82]. Indole-3-acetic acid is another IAA which was found in the skin of *Bufo alvarius* [25]. Several pharmacological activities of indole-3-acetic acid was reported, including anti-inflammatory, antipyretic [83], antifungal [84], hypoglycemic [85], and anticancer [86].

## 5. Conclusions

The studies of toad toxins in the past years have demonstrated new perspectives for their pharmaceutical use, not only for treating cardiac failure, but also for other therapeutic purposes, for example, as anti-inflammatory, immunoregulatory, and anticancer compounds. The understanding of the chemical basis of toad toxins has provided the basis to develop new therapeutic agents from different species of toads. Primarily, due to the environmental pollution in China, there is currently a shortage of toad resources in the pharmaceutical industry, while toads in some countries are becoming natural disasters that need to be managed, such as the cane toad in Australia. Therefore, the development of toad medicines from different resources is acutely needed.

Currently, several fundamental questions remain to be resolved to fully reveal the potential use of toad secretions. Although a number of in vitro studies have been done by researchers on Chansu, Huachansu, and single compounds, regarding their effects and mechanisms, the in vivo and clinical studies are still very limited. Thus, it is important to perform more animal studies to decode the potential of toad toxins in treating various diseases, such as cancer. Additionally, digitalis toxicity has always been a main concern for scientists in using toad medicines in patients [87]. Several reports have shown cardiotoxicity caused by bufadienolides [88]. Therefore, there is an urgent need to study the toxicities and the maximum tolerated dose of toad toxins. Beyond that, how to reduce the side effects is the next step to be considered. The knowledge of TCM formulas may provide us with some good points for resolving this problem. Chansu is generally used as a recipe with other herbs, to prepare formulations such as She Xiang Bao Xin Wan for the treatment of cardiovascular diseases, or Mei Hua Dian She Wan and Liu Shen Wan for the treatment of inflammatory diseases. Another herb, by the name of Bezoar Bovis, was frequently used in preparations of Chansu (Table 3). There has been a study showing that the use of Bezoar Bovis protects Chansu-induced acute toxicity in mice. Further study has shown that the taurine derived from Bezoar Bovis also prevented Chansu- or bufalin-caused cardiotoxicity, and reduced the mortality in animal models [75,76]. Other studies have also indicated that the use of nanoparticles may help improve antitumor activity while reducing the side effects of toad medicines [89].

Moreover, the quality control of using natural products as therapeutic agents has always been a high concern for researchers. Some chemistry studies have indicated that the same species of toads obtained from different geographical regions, and under different conditions of weather, time, and other environmental factors, will result in an impact on their chemical compositions. Therefore, chemical analysis to quantify the various compounds present, and quality control to ensure the consistency of preparations in the study, are crucial issues that need to be considered [2,32].

Taken together, toad toxins from different species have a promising role in treating various diseases. However, the molecular mechanisms, drug safety, and the demand for quality control need to be resolved in future studies. No doubt though, the application of toad toxins as novel therapeutic agents will contribute to the world in many different aspects in terms of scientific research, pharmaceutical industry, environmental protection, and economic growth.

## Figures and Tables

**Table 1:** The identification of significant bioactive compounds in different species of toads.

Name	Classification	Formula	Species of Toad			
			*B. b. gargarizans*	*B. marinus*	*B. alvarius*	*B. melanosticus*
Bufalin	Bufadienolides	C_24_H_34_O_4_	+	+	+	+
Cinobufagin	Bufadienolides	C_26_H_34_O_6_	+	−	−	−
Arenobufagin	Bufadienolides	C_24_H_32_O_6_	+	+	+	+
Gamabufotalin	Bufadienolides	C_24_H_34_O_5_	+	−	+	+
Telocinobufagin	Bufadienolides	C_24_H_34_O_5_	+	+	+	+
Marinobufagin	Bufadienolides	C_24_H_32_O_5_	+	+	+	+
Bufotenine	Indolealkylamine	C_12_H_16_N_2_O	+	+	+	+
Bufotenidine	Indolealkylamine	C_13_H_18_N_2_O	+	−	−	+
Dehydrobufotenine	Indolealkylamine	C_12_H_14_N_2_O	+	+	−	+
Bufothionine	Indolealkylamine	C_12_H_15_N_2_O_3_S	+	+	+	−
5-methoxytryptamine	Indolealkylamine	C_11_H_14_N_2_O	−	+	+	−
Indole-3-acetic acid	Indolealkylamine	C_10_H_9_NO_2_	−	−	+	−

+: Present; −: Not present.

**Table 2:** Molecular targets of bufadienolides found in a wide range of preclinal models.

Compound	Experimental Models	Molecular Targets	References
Bufalin	In vitro/In vivo	Macrophages, eosinophils, lymphocytes, and neutrophils and cytokines including IL-4, IL-5, and IL-13, NF-κB	[44]
	In vivo	iNOS, COX-2, IL-1β, IL-6, TNF-α, NF-κB	[45]
	In vitro	MMP-2, MMP-9, PI3K, AKT, NF-κB	[46,47]
	In vivo	Bax	[49]
	In vivo	AKT/GSK3β/β-catenin/E-cadherin	[50]
	In vivo	PTEN/phosphate-PTEN, AKT/phosphate-AKT, Bad, Bcl-xl, Bax, or Caspase-3	[51]
Cinobufagin	In vitro/In vivo	Caspase-3, hypoxia-inducing factor-1 alpha	[53]
	In vivo	Cortactin	[54]
	In vitro	Notch pathway	[55]
Arenobufagin	In vitro/In vivo	p53 pathway	[56]
	In vitro/In vivo	Bax/Bcl-2, PI3K/Akt/ mTOR pathway. ADP-ribose polymerase, light chain 3-II	[57]
	In vivo	β-catenin	[58]
	In vitro/In vivo	VEGFR-2 pathway	[59]
Gamabufotalin	In vivo	VEGFR-2 pathway	[60]
	In vitro/In vivo	c-Myc	[61]
	In vitro/In vivo	IKKβ, NF-κB, COX-2, p65	[62]
Telocinobufagin	In vitro	CD4, CD8, IL-2, IL-12, IFN-γ, TNF-α, IL-4	[63]

**Table 3:** Some classic recipes contain Chansu in traditional Chinese medicine (TCM).

Recipe Name	Treatment Purpose	Main Ingredients
Liu Shen Wan	Inflammatory and infectious diseases, etc.	Chansu, Pearl Powder, Bezoar Bovis, Musk, Realgar, and Bornel
She Xiang Bao Xin Wan	Congestive heart failure	Chansu, Ginseng, Bezoar Bovis, Musk, Cinnamon, Liquidambar, and Borneol
Mei Hua Dian She Wan	Relieves swelling and pain	Chansu, Borneol, Cinnabar, Myrrh, Bezoar Bovis, Borax, Frankincense, Musk, Draco Seed, Realgar, Bear Gall, Blood Dracon, Pearl Powder, and Cinnabar
